# Supplementary material for: Effect of Wakame and Carob Pod Snacks on Non-Alcoholic Fatty Liver Disease
Source: Nutrients. 2019 Jan 4;11(1):86. doi: 10.3390/nu11010086 (PMC6356417; doi:10.3390/nu11010086)
Supplement: Supplementary file 1 [file nutrients-11-00086-s001.pdf]

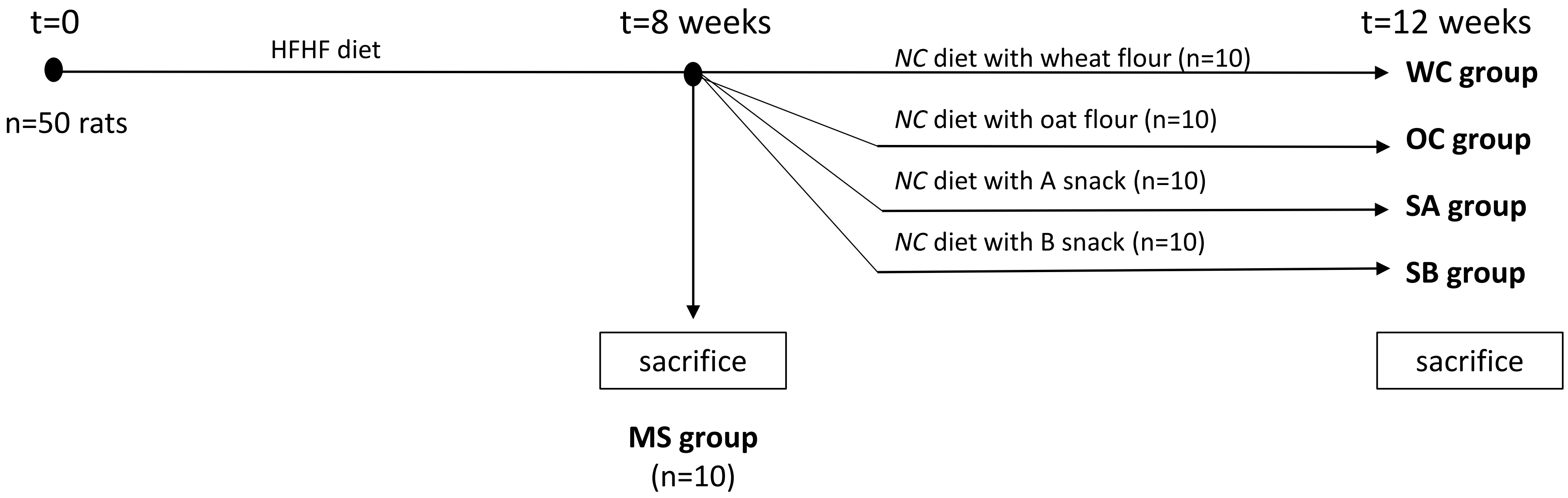

Supplementary figure 1: Flow chart of the experimental design. *HFHF*: High-fat high-fructose. *NC*: Normal-caloric. MS: Metabolic síndrome. WC: Wheat control. OC: Oat control. SA: Snack A. SB: Snack B.
